# Microbiomes Associated With Foods From Plant and Animal Sources

**DOI:** 10.3389/fmicb.2018.02540

**Published:** 2018-10-23

**Authors:** Karen G. Jarvis, Ninalynn Daquigan, James R. White, Paul M. Morin, Laura M. Howard, Julia E. Manetas, Andrea Ottesen, Padmini Ramachandran, Christopher J. Grim

**Affiliations:** ^1^Center for Food Safety and Applied Nutrition, U.S. Food and Drug Administration, Laurel, MD, United States; ^2^Resphera Biosciences, Baltimore, MD, United States; ^3^Office of Regulatory Affairs, Northeast Food and Feed Laboratory, U.S. Food and Drug Administration, Jamaica, NY, United States; ^4^Center for Food Safety and Applied Nutrition, U.S. Food and Drug Administration, College Park, MD, United States

**Keywords:** microbiome, 16S rRna, food, metagenomics, produce, spices, seafood

## Abstract

Food microbiome composition impacts food safety and quality. The resident microbiota of many food products is influenced throughout the farm to fork continuum by farming practices, environmental factors, and food manufacturing and processing procedures. Currently, most food microbiology studies rely on culture-dependent methods to identify bacteria. However, advances in high-throughput DNA sequencing technologies have enabled the use of targeted 16S rRNA gene sequencing to profile complex microbial communities including non-culturable members. In this study we used 16S rRNA gene sequencing to assess the microbiome profiles of plant and animal derived foods collected at two points in the manufacturing process; post-harvest/pre-retail (cilantro) and retail (cilantro, masala spice mixes, cucumbers, mung bean sprouts, and smoked salmon). Our findings revealed microbiome profiles, unique to each food, that were influenced by the moisture content (dry spices, fresh produce), packaging methods, such as modified atmospheric packaging (mung bean sprouts and smoked salmon), and manufacturing stage (cilantro prior to retail and at retail). The masala spice mixes and cucumbers were comprised mainly of *Proteobacteria*, *Firmicutes*, and *Actinobacteria*. Cilantro microbiome profiles consisted mainly of *Proteobacteria*, followed by *Bacteroidetes*, and low levels of *Firmicutes* and *Actinobacteria*. The two brands of mung bean sprouts and the three smoked salmon samples differed from one another in their microbiome composition, each predominated by either by *Firmicutes* or *Proteobacteria*. These data demonstrate diverse and highly variable resident microbial communities across food products, which is informative in the context of food safety, and spoilage where indigenous bacteria could hamper pathogen detection, and limit shelf life.

## Introduction

You are what you eat. This idea, that the food that you consume controls your health, is not novel, and may be attributed to an early monograph on gout by Brillat-Savarin (2010). In recent years, efforts led by the Human Microbiome Project and the MetaHIT consortium have greatly expanded our knowledge of the microbiomes associated with the human host, especially the gastrointestinal (GI) tract (Qin et al., 2010; Human Microbiome Project Consortium, 2012a,b). That knowledge appears to have important consequences for individual human health. This paper explores food microbiomes to identify bacteria that are associated with foods and therefore have the potential to colonize our gastrointestinal tract. These resident cohorts of bacteria on our food may also be a factor in food safety, affecting our ability to detect and identify pathogens during foodborne outbreaks, and contribute to the general transmission of bacteria between the environment and the human body.

More specifically, surveys of microbial populations in the human gut led Arumugam and colleagues to describe three distinct “Enterotypes” or microbial communities that can differ between individual human hosts (Arumugam et al., 2011; Costea et al., 2018). Enterotypes seem to be controlled by fetal microbiome composition, blood type, and the metabolic potential of the associated microbiome. Once established, the most likely source of new microbes joining our GI microbiome is the food we eat: each food stuff and commodity we consume likely contains a microbiome that passes through our bodies while nutritional ingredients and components are digested. It is unclear how much of this inoculum successfully passes through our gastric barrier, reaches our intestine and colon, resulting in colonization. Foods such as fresh produce that are often consumed raw with no kill step also have the potential to alter the microbial composition of our GI tract and, if they harbor pathogens, can cause illness. Thus, characterizing food microbiomes may provide reference points for understanding which bacteria are beneficial, how frequently our GI tract is exposed to foodborne pathogens, and how these bacterial communities interact with one another. Current methods to detect foodborne pathogens such as *Salmonella*, *Escherichia coli*, and *Listeria* rely on culturing these organisms directly from food, which requires at least 1 week for the isolation of a presumptive positive colony, followed by 2–4 days for culture confirmation. These methods are complex and challenging due to the wide variety of food matrices implicated in outbreaks and the presence of many different bacterial species residing in foods that grow along with adulterating organisms and may compromise pathogen detection.

Recent studies employing microbiome profiling have revealed that the microflora indigenous to tomatoes, cilantro, spinach, mung bean sprouts, and ice cream, can affect our ability to detect *Salmonella*, *Listeria*, and *E. coli* in these foods primarily because the incursion of a foodborne pathogen typically occurs at a very low abundance compared to the resident microflora (Ottesen et al., 2013; Jarvis et al., 2015; Leonard et al., 2015, 2016; Margot et al., 2016; Ottesen et al., 2016). For example, detection of Shiga Toxigenic *Escherichia coli* (STEC) in spinach is hampered by non-pathogenic *E. coli* and detection of *Salmonella* is greatly reduced in foods, such as cilantro, that also harbor closely related species such as *Enterobacter* and *Citrobacter* (Leonard et al., 2015; Daquigan et al., 2016; Grim et al., 2017). These studies suggest that both bacterial load and the composition of the indigenous microbiota can influence our ability to detect foodborne pathogens. Identifying the intrinsic bacterial biomass in foods and how those intrinsic bacterial communities are different from commodity to commodity is a fundamental first step for assessing the sensitivity and specificity of microbiome profiling for detecting pathogens in foods.

Analysis of food microbiomes can also provide insights into food quality by revealing the presence of spoilage associated microorganisms. Microbial spoilage is the most common cause of food spoilage which is considered as any change in food that renders it unacceptable to a consumer including sensory characteristics such as changes in color, texture, and development of off odors and colors (Gram et al., 2002). Some examples of bacteria commonly associated with food spoilage include *Pseudomonas* species in plant and animal derived foods, *Xanthomonas* in fresh produce, and *Shewanella* in chilled fish and meat (Blackburn, 2006). *Enterobacteriaceae* are natural inhabitants of many food plants and animals providing numerous opportunities for introduction into the food supply. For example, the production of pectic enzymes by *Erwinia* species result in soft rot of fresh vegetables, and the production of volatile compounds by *Serratia* and *Proteus* species results in the spoilage of dry cured meats (Blackburn, 2006). Food spoilage caused by Lactic Acid Bacteria (LAB) is influenced by intrinsic and extrinsic factors such as low pH and temperature or types of food packaging such as modified atmospheric packaging (MAP) that lower oxygen levels. *Lactobacillus* and *Leuconostoc* species have been associated with spoilage of vacuum packaged meat and fish, low pH foods such as, ketchup and salad dressings, and cheeses (Blackburn, 2006). Additional spoilage- associated bacteria include spore-formers such as *Bacillus*, and other genera such as *Acinetobacter*, *Flavobacterium*, *Psychrobacter*, and *Brochothrix* that are responsible for spoilage of particular food groups. For example, *Acinetobacter* have been isolated from soil, water, sewage, milk, vegetables, and chicken. *Flavobacterium* species, also commonly found in the environment in soil and water, prefer chilled foods such as milk, raw meats, and wild and farmed fresh-water fish (Blackburn, 2006).

The purpose of this study was to define the baseline microbiomes, employing 16S rRNA gene sequencing, of the plant-derived commodities cilantro, mung bean sprouts, cucumbers, and masala spice mixes, sourced from two points in the food supply chain. In order to have a non-plant-derived commodity for comparison, we analyzed three smoked salmon samples to demonstrate the value of microbiome profiling for assessing the microbiota associated with the smoking process. These commodities represent the global nature of our food supply and have been linked to foodborne outbreaks in the past. For example, a 2007 – 2009 survey of imported spices determined that the mean prevalence of *Salmonella* was 0.066 (95% CI 0.057–0.076) and included a variety of serotypes some with antimicrobial resistance profiles (Van Doren et al., 2013). Herbs and spices contaminated with *Salmonella* have resulted in numerous outbreaks including one in Germany due to contaminated paprika and another in the United States caused by contaminated cilantro (Lehmacher et al., 1995; Campbell et al., 2001). Since 2013, four *Salmonella* outbreaks have been attributed to domestic and imported cucumbers and epidemiological evidence identified four serotypes (Poona, Newport, Saint Paul, and Oslo) responsible for these outbreaks (Angelo et al., 2015; Bottichio et al., 2016). Seed and bean sprouts were linked to 33 outbreaks from 1998 through 2010 (Dechet et al., 2014); despite rigorous guidelines to reduce contamination these continue to be implicated in outbreaks of *Salmonella* and *Escherichia coli* (Mohle-Boetani et al., 2009; CDC, 2013; Bayer et al., 2014). Smoked salmon products have historically been associated with *Salmonella* and *Listeria* outbreaks (Rorvik, 2000; Friesema et al., 2014).

## Materials and Methods

### Foods Used in This Study

The Department of Agriculture and Rural Development in Lansing Michigan (MDARD) collected seven cilantro samples prior to retail distribution, in January, February, and March of 2015 (Supplementary Table 1). Six replicates of six MDARD cilantro samples (CIL27A, CIL27B, CIl27C, CIL28A, CIL28C, and CIL28D) and three replicates of one MDARD cilantro sample (CIL4Z) were sequenced. An additional set of cilantro samples and two brands of mung bean sprouts were purchased from a local retail store in April of 2015. The retail cilantro samples were combined into a homogenous composite and eighteen replicates (CILSB1 thru CILSB18) were sequenced. Three replicates of mung bean sprout brand A (SPA), and nine replicates of mung bean sprout brand B (SPB) were sequenced (Supplementary Table 1). Seven cucumber samples (CUC2, CUC3, CUC4, CUC5, CUC7, CUC8, and CUC9) were collected from a farm in New Jersey in November of 2014 and sequenced individually. We also sequenced Chicken Masala (MA), Fish Masala (MB), Meat Masala (MC), Egg Masala (MD), and Garam Masala (ME) spice mixtures. These masala spices were mixtures of 8–17 individual spices that were imported from India in November of 2014; two replicates of each spice mixture were sequenced (Supplementary Table 2). Three smoked salmon samples, SmkSalD, SmkSalE, and SmkSalF, were obtained from the California Department of Public Health, Richmond, CA, United States in January 2016, and two replicates of each were sequenced.

Culture enrichment media was used as “wash” media to remove the microbiome from each food as follows: twenty-five grams of each food sample was aseptically transferred to separate, sterile Whirlpak bags (Nasco, Fort Atkinson, WI, United States) and mixed with a sterile culture medium at a 1:10 (w/v). Cilantro samples were rinsed with modified Buffered Peptone Water (mBPW). Mung bean sprouts and smoked salmon samples were rinsed with Buffered *Listeria* Enrichment Broth. Cucumbers, and masala spice mixes were rinsed with Lactose Broth, and Trypticase Soy Broth, respectively. All samples were hand massaged for 2 min at room temperature in their respective broth to dislodge resident bacteria. Aliquots (15 ml) were removed and centrifuged at 7,100 rcf for 30 min and bacterial pellets were stored at -20°C.

### Preparation of Genomic DNA

Genomic DNA was prepared from the bacterial pellets of the microbiomes of masala spice mixes, cilantro, cucumber, mung bean sprouts, and smoked salmon using the DNAeasy Blood and Tissue (Qiagen, Germantown, MD, United States) protocol on the QIAcube (Qiagen, Germantown, MD, United States) automated sample preparation instrumentation.

### 16S rRNA Gene Amplicon Library Preparation and Sequencing

16S rRNA gene library preparation, sequencing, data processing and analyses were performed as described by Daquigan et al. (2016). The PCR primers used in this study targeted the V1 – V3 hypervariable regions (508 bp) of the 16S rRNA gene. Amplicon libraries were sequenced in six separate MiSeq runs multiplexed at 96 samples per run except for the fifth and sixth MiSeq runs, which only contained 60 and 86 samples, respectively (Supplementary Table 3). To normalize across samples prior to downstream analyses, the 16S rRNA gene sequence profiles were subsampled to an even level of coverage resulting in 10,000 16S rRNA sequence reads for each cilantro, mung bean sprout, cucumber, and masala spice mix sample, and 30,000 16S rRNA sequence reads for each smoked salmon sample. High-quality 16S rRNA gene sequence reads were assigned to a taxonomic lineage using Resphera Insight (Resphera Biosciences, Baltimore, MD, United States; Daquigan et al., 2016; Abernethy et al., 2017; Drewes et al., 2017; Grim et al., 2017). Using these methodologies our sensitivity for cilantro, cucumbers, mung bean sprouts, cucumbers, and masala spice mixes with 10,000 sequence reads per sample we have a 95% chance of observing a pathogen with at least 0.03% abundance (Grim et al., 2017). In smoked salmon, with 30,000 sequence reads per sample, we have a 95% chance of observing a pathogen with at least 0.01% abundance (Grim et al., 2017).

### Database Submission

16S rRNA gene sequence data from this study has been submitted to the FDA MetaGenomeTraker Project (NCBI Accession PRJNA390622).

## Results

### Bacterial Species Diversity Varied by Commodity

The species richness in plant-derived foods was highest in the masala spice mixes with observed species ranging from 968 to 1097 (Supplementary Table 1). Mung bean sprouts, cilantro and cucumber observed species ranged from 226 to 340 (mung bean sprouts), 216 to 573 (cilantro), and 227 to 423 (cucumbers) (Supplementary Table 1). The Simpson reciprocal index measures of diversity corroborated that the species richness was highest in the masala spice mixes and lowest in the mung bean sprouts (Supplementary Table 1) (Simpson, 1949). The species richness of the smoked salmon samples was lower than the plant derived foods with observed species ranging from 89 to 181 and, as expected, the alpha diversity in these samples was low (Supplementary Table 1).

**FIGURE 1:**
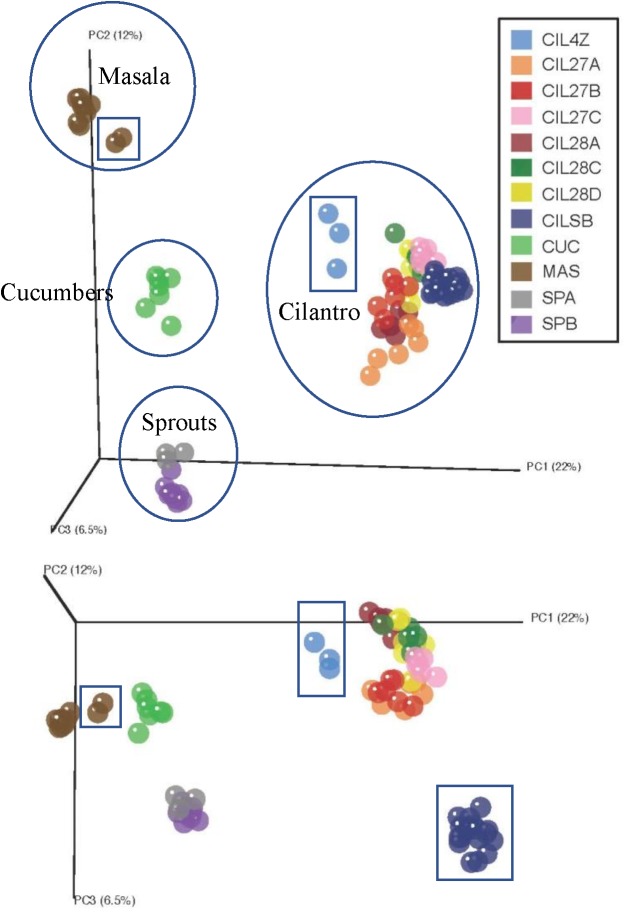
Principal coordinates analysis reveals clustering by commodity type. Each food is circled and squares indicate garam masala (brown), MDARD cilantro CIL4Z (light blue), and retail cilantro CILSB (dark blue).

Unweighted UniFrac distance measures revealed striking variations among the microbiota of the plant-derived foods (Figure 1) (Lozupone and Knight, 2005). The first coordinate, accounting for 22% of the variation in beta-diversity among all food samples, clearly separates our samples into four groups corresponding to the type of food: cucumbers, cilantro, mung bean sprouts, and masala spice mixes (Figure 1). The second and third coordinates, representing 12 and 6.5% of the beta-diversity, further separate the cilantro, mung bean sprout, and masala spice microbiota indicating differences even within a single food type (Figure 1).

**FIGURE 2:**
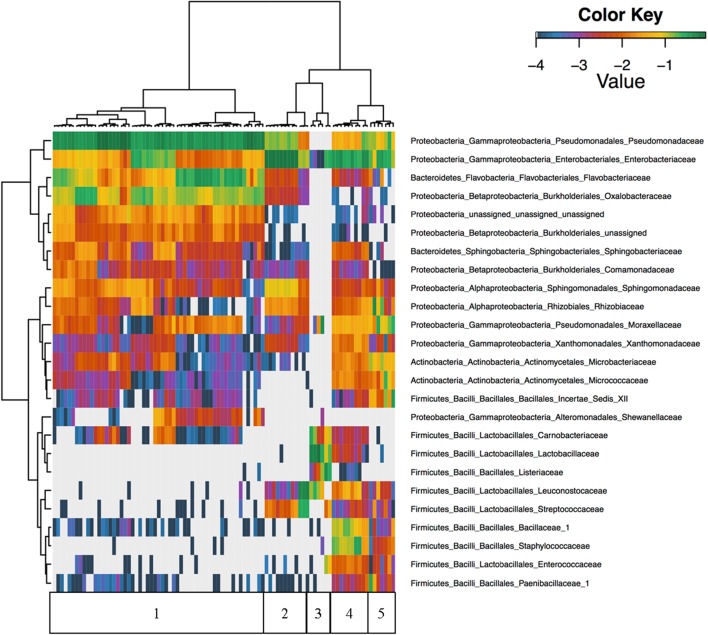
Unsupervised hierarchical clustering of the top 25 families across all samples. Values reflect proportional abundances. 1 = cilantro, 2 = Mung bean sprouts, 3 = Smoked Salmon, 4 = Masala spice mixes, 5 = Cucumbers.

Unsupervised hierarchical clustering of all foods revealed that *Firmicutes* were nearly absent in cilantro with proportional abundances of <1% in all but nine of the cilantro samples, and abundances in these nine samples only reached 2.3% (Figure 2). Furthermore, *Pseudomonadaceae*, *Enterobacteriaceae*, *Flavobacteriaceae*, and *Oxalobacteraceae* were the predominant taxa driving beta-diversity (Figure 2). For example, we observed that proportional abundances of *Enterobacteriaceae* vary greatly within each commodity; these are generally the lowest in cilantro (34 to 1%), and smoked salmon (7% to 0%) and the highest in SPB (69%) (Figure 2 and Supplementary Figure 1). *Oxalobacteraceae* and *Flavobacteriaceae* are nearly absent in all commodities, except cilantro, in which proportional abundances vary from 1% in CIL4Z to 29% in CIL28C for *Oxalobacteraceae* and 2% in CIL4Z to 34% in CILSB for *Flavobacteriaceae* (Figure 2 and Supplementary Figures 2, 3). Furthermore, cilantro had the highest proportional abundances of *Pseudomonadaceae* among all foods (44–79%) while the smoked salmon harbored the lowest abundances of these members (<1%) (Figure 2 and Supplementary Figure 4).

We observed high abundances of *Leuconostocaceae* and *Streptococcaceae* in SPA, which include members associated with food spoilage, but not in SPB. *Leuconostocaceae* proportional abundances are also high in smoked salmon, which also harbored high abundances of spoilage associated *Listeriaceae*, *Lactobacillaceae*, and *Carnobacteriaceae* (Figure 2). Additionally, of the top 25 families identified across all foods, 19 were absent from the smoked salmon samples (Figure 2). Finally, the microbiota found on the masala spices included high proportional abundances of *Bacillaceae*_1 and *Staphylococcaceae* (Figure 2).

### Masala Spice Mixes Had the Highest Species Diversity

**FIGURE 3:**
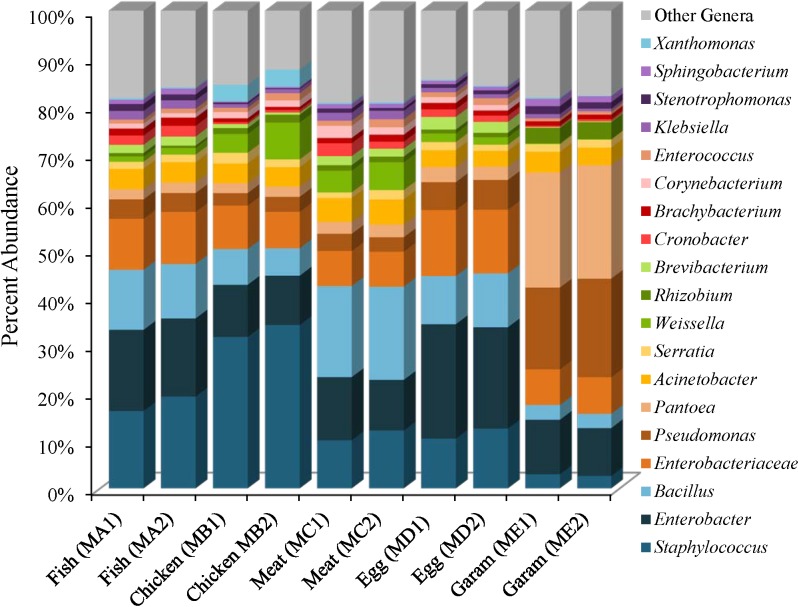
Genus level proportional abundances (≥1%) of masala spice mixes. Two replicates of each spice mixture were sequenced.

The five masala spice mixes, Chicken, Fish, Meat, Egg, and Garam, exhibited a potential correlation between the number of spices in each mix and levels of species diversity (*P* = 0.08, Pearson coefficient = 0.58). Specifically, the Meat masala, which contained 17 ingredients, had the highest diversity and the Fish masala, which contained the least number of ingredients had the lowest species diversity (Supplementary Table 2). Bacterial communities found in masala spice mixes consisted primarily of *Proteobacteria* (41–84%) and *Firmicutes* (9–54%), followed by *Actinobacteria* (5–12%) and *Bacteroidetes* (0.41–2%), although the Garam masala had higher abundances of *Proteobacteria* and lower abundances of *Firmicutes* than the rest of the spice mixtures (Supplementary Table 4). The difference in the Garam masala composition was due to higher proportional abundances of *Pseudomonas* (19%) and *Pantoea* (24%) members in conjunction with a lower proportional abundance of *Staphylococcus*; 3% in Garam as compared to 11–39% in the remaining four masala spice mixes (Figure 3 and Supplementary Figures 4, 5). The fennel seeds which are unique to the Garam mix, may account for the higher abundances of *Pseudomonas* and *Pantoea* in this spice mix (Supplementary Table 3). Unassigned *Enterobacteriaceae* Operational Taxonomic Units (OTUs) were observed in all of the spice mixes; the Egg masala had the highest proportional abundances (10%) which, likely represent additional *Enterobacter* species (Figure 3 and Supplementary Table 5). *Bacillus* species were also identified in all masala spices at proportional abundances as high as 13% in the Meat masala (Figure 3 and Supplementary Table 5).

### Cilantro Microbiomes Vary by Source

The cilantro microbiota consists primarily of *Proteobacteria* (54–99%), followed by *Bacteroidetes* (0.83–46%), *Actinobacteria* (0.03–5%) and *Firmicutes* (0–2%) (Supplementary Table 4). The Simpson reciprocal and Shannon diversity indexes for cilantro samples ranged from 3.97 to 33.89 and 3.81 to 6.34, respectively and the subsamples had similar levels of diversity (Supplementary Table 2). For example, the highest alpha diversity was observed in CIL27C, which also harbored the highest number of observed species.

**FIGURE 4:**
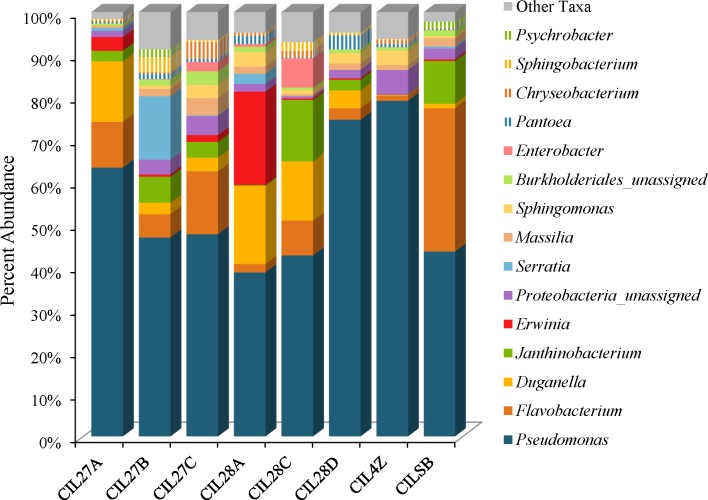
Genus level average proportional abundances (≥1%) of MDARD and retail cilantro samples. Proportional abundances were average for six replicates of MDARD samples CIL27A, CIL27B, CIl27C, CIL28A, CIL28C, and CIL28D and three replicates of MDARD sample CIL4Z. The proportional abundances for the retail cilantro samples CILSB1 thru CILSB18 were averaged.

Weighted UniFrac analysis resulted in three distinct cilantro clusters; (1) the retail samples, (2) 36 of the MDARD samples and (3) CIL4Z, also an MDARD sample (Figure 1). The presence of *Flavobacterium* at proportional abundances of 34% in the retail cilantro samples, CILSB and their absence in the MDARD samples led to distinct clustering of these groups (Figures 1, 4 and Supplementary Figure 3). In fact, CILSB, the retail cilantro, was the only food in this study that contained *Flavobacterium* (Figure 4 and Supplementary Figure 3). These retail cilantro samples harbored at least seven distinct *Flavobacterium* OTUs (Supplementary Table 5). The MDARD cilantro sample CIL4Z contained high proportional abundances of *Pseudomonas* (79%) that accounted for most of the *Proteobacteria* in this cilantro sample (Figure 4 and Supplementary Figure 4). Additionally, two members of the *Oxalobacteraceae* family, *Janthinobacterium* and *Duganella*, were absent in the microbiota of CIL4Z further distinguishing them from the rest of the cilantro samples (Figure 4, Supplementary Figure 2, and Supplementary Table 5).

Although cilantro harbored the lowest proportional abundances of *Enterobacteriaceae* in this study, MDARD samples CIL28A and CIL27B had notable abundances of *Erwinia* (14–29%) and *Serratia* (11–19%) (Figure 4, Supplementary Figure 1, and Supplementary Table 5).

### Cucumber Microbiomes

**FIGURE 5:**
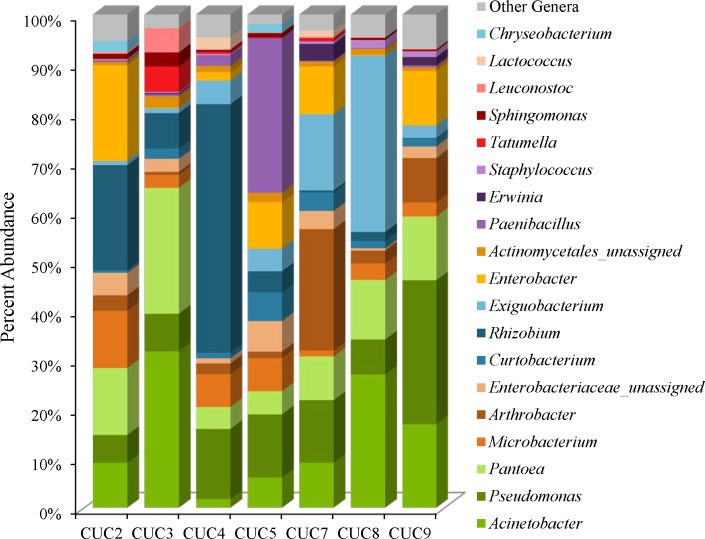
Genus level proportional abundances (≥1%) of seven cucumber samples.

The cucumber microbiota is comprised of *Proteobacteria* (45–85%), followed by *Firmicutes* (2–40%), *Actinobacteria* (8–31%), and *Bacteroidetes* (0–2%) (Supplementary Table 4). The Simpson reciprocal and Shannon alpha-diversity indices range from 7.9 (CUC4) to 34.1 (CUC9) for the Simpson reciprocal and 4.2 (CUC8) to 6.3 (CUC9) for the Shannon index (Supplementary Table 1). Although the weighted UniFrac clustering results indicate a distinct microbiota for cucumbers, we did observe variation in the abundances of predominant community members (Figures 1, 2, 5). For example, *Acinetobacter* were observed on all cucumbers, at proportional abundances ranging from 2 to 32% (Figure 5 and Supplementary Table 5). *Rhizobium* proportional abundances ranged from 50% in CUC4 to less than 0.5% in CUC7 and CUC9 (Figure 5 and Supplementary Table 5). Additionally, each cucumber harbored *Pantoea* at abundances of 4–25%, *Microbacterium* and *Curtobacterium* species (Figure 5, Supplementary Figure 5, and Supplementary Table 5). Like the masala spice mixes, unassigned *Enterobacteriaceae* OTUs were observed in four of the cucumber samples at proportional abundances ranging from 3% to 5%, most likely represent *Enterobacter* (Figure 5 and Supplementary Table 5). There were also a few distinguishing genera present at relatively high proportional abundances in some cucumbers. For example, *Paenibacillus* were observed at abundances of 31% in CUC5 and two *Exiguobacterium* OTUs were observed in cucumbers at proportional abundances of 14 and 20% in CUC8, and 5 and 9% in CUC7 (Figure 5 and Supplementary Table 5). In the *Actinobacteria* phyla, we observed *Arthrobacter* in CUC7 at proportional abundances of 25%, and 12% of these OTUs were classified as *Arthrobacter defluvii* (Figure 5 and Supplementary Table 5). Finally, some cucumbers harbored spoilage-associated bacteria such as *Lactococcus*, in CUC4 (2%) and CUC7 (1%), and *Leuconostoc* present in CUC3 (5%) (Figure 5 and Supplementary Table 5).

### Mung Bean Sprout Microbiomes Reveal Indicators of Spoilage

**FIGURE 6:**
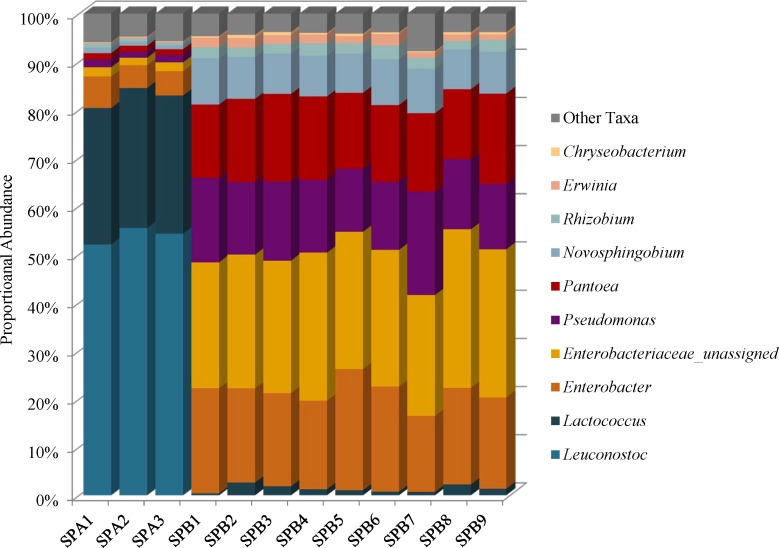
Genus level proportional abundances (≥1%) of three replicates of mung bean sprout brand A (SPA) and nine replicates of mung bean sprout B (SPB).

Species diversity in the mung bean sprouts were lower in SPA (brand A) than SPB (brand B). The Simpson reciprocal index ranges were 6.44–7.21 in SPA and 11.55–17.37 in SPB, and the Shannon indices ranged from 3.65 to 3.89 in SPA and 4.94 to 5.34 SPB (Supplementary Table 2). *Firmicutes* (81–85%) predominated in SPA followed by *Proteobacteria* (15–19%), *Bacteroidetes* (0.11–0.14%), and *Actinobacteria* (0–0.01%) while the SPB, harbored mostly *Proteobacteria* (96–99%) with proportionately less *Firmicutes* (0.42–2.72%); the *Bacteroidetes* (0.36–0.79%), and *Actinobacteria* (0–0.06%) abundances were similar to SPA (Supplementary Table 4). These differences were due to higher proportional abundances of spoilage-associated *Leuconostoc* (54%) and *Lactococcus* species (29%) in SPA that were present at very low levels in SPB, 0.04 and 1.3%, respectively (Figure 6 and Supplementary Table 5). Although the SPA microbiomes were less complex, we did identify 3% proportional abundances of *Enterobacteriaceae incertae sedis* OTUs, which likely represent *Kosakonia cowanii*, a recently classified species previously known as *Enterobacter cowanii* (Supplementary Table 5) (Brady et al., 2013).

Unassigned *Enterobacteriaceae* (27%), *Enterobacter* (20%), *Pseudomonas* (16%), and *Pantoea* (16%) predominate in SPB, and these samples harbor the highest proportional abundances of *Enterobacteriaceae* of all foods in this study (Figure 6 and Supplementary Figure 1). The unassigned *Enterobacteriaceae* in SPB are mostly likely members of the *Enterobacter* genera (Figure 6 and Supplementary Table 5).

### Smoked Salmon Microbiota Contains Organisms Associated With Spoilage and Preservation

To highlight potential differences between plant and animal derived commodities, we sequenced three smoked salmon samples. Smoked salmon species diversity was lower than plant derived food species diversity; Simpson reciprocal and Shannon diversity indices ranged from 2.47 to 7.90 and 1.34 to 2.65, respectively (Supplementary Table 2). The microbiomes were comprised almost entirely of *Proteobacteria* and *Firmicutes* at proportional abundances that varied between the three samples such that SmkSalD harbored extremely low levels of *Proteobacteria* (0.41 and 0.15%) as compared to SmkSalE (76 and 51%) and SmlSalF (50 and 25%) (Supplementary Table 6). Likewise, the *Firmicutes* abundances varied from 99% in SmkSalD, 24 to 49% in SmkSalE and 50 to 75% in SmkSalF (Supplementary Table 6).

**FIGURE 7:**
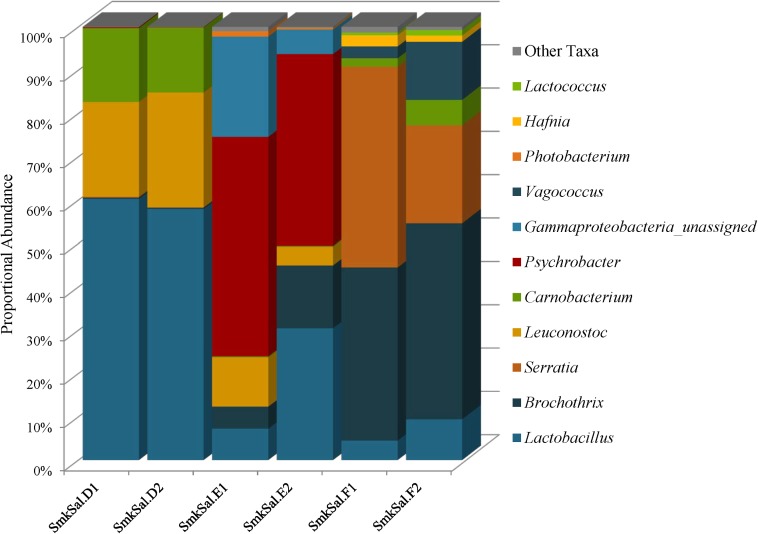
Genus level proportional abundances (≥1%) of three smoked salmon samples. Two replicates of each sample were sequenced.

The *Firmicutes* identified in smoked salmon: *Lactobacillus*, *Brochothrix*, *Carnobacterium*, *Leuconostoc*, *Vagococcus*, and *Lactococcus*, are commonly associated with spoilage of refrigerated high-protein foods, such as meat and fish (Figure 7 and Supplementary Table 6). *Lactobacillus* abundances were highest in the SmkSalD replicates (57 and 55%) and dropped as low as 0.48% in SmkSalF (Figure 7 and Supplementary Table 6). *Brochothrix* predominated in SmkSalF with proportional abundances of 39 and 44% in F1 and F2, respectively (Figure 7 and Supplementary Table 6). SmkSalE also harbored *Brochothrix* but at much lower proportional abundances, 5% in E1 and 13% in E2; SmkSalD was almost deplete of this taxa with abundances less than 1% (Figure 7 and Supplementary Table 6). Two OTUs of *Carnobacterium* were observed in smoked salmon at levels that suggest competition. One *Carnobacterium* OTU reached abundances of 5% in SmkSalD and SmkSalF but abundances in SmkSalE were <1%, and abundances of the second OTU were highest in SmkSalD reaching 8% in D1 and 6% in D2, while remaining very low (<1%) in SmkSalE and SmkSalF (Figure 7 and Supplementary Table 6). *Leuconostoc* abundances were highest in SmkSalD (26%), followed by SmkSalE, and notably absent in SmkSalF. Two *Vagococcus* OTUs were observed in SmkSalF at proportional abundances of 2 and 7% in F1 and F2, respectively (Figure 7 and Supplementary Table 6).

*Proteobacteria* were only observed in SmkSalE and SmkSalF, and the members of this phyla were very different from those observed in plant derived foods. For example, *Psychrobacter* were observed in SmkSalE at proportional abundances of 22% (E1) and 23% (E2) (Figure 7 and Supplementary Table 6). SmkSalE also harbored unassigned *Gammaproteobacteria* OTUs at relatively high proportional abundances of 23% in E1 and 6% in E2, suggesting a novel community member or OTUs that could not be resolved beyond the class level with our analysis tool (Supplementary Table 6). The *Proteobacteria* in SmkSalF were distributed among three *Serratia* OTUs (Figure 7 and Supplementary Table 6). SmkSalF1 also harbored *Hafnia* OTUs at proportional abundances of 2% (Figure 7 and Supplementary Table 6).

### Unique and Shared Food Microbiome Members

Some genera were distinct to plant and animal derived foods. For example, *Sphingomonas*, *Pseudomonas*, *Pantoea*, *Acinetobacter*, and *Erwinia* were present in all the plant foods; the latter four cause food spoilage (Supplementary Table 7). *Hafnia* and *Morganella*, both commensals of the human gastrointestinal tract, *Brochothrix*, linked to meat spoilage, and *Photobacterium*, a bioluminescent marine bacteria of the *Vibrionaceae* family, were unique to smoked salmon. The masala spice mixes, which had the highest alpha diversity in our study, harbored 19 unique genera including food spoilage associated bacteria such as *Bacillus*, and *Cronobacter* which are known foodborne pathogens, and *Pediococcus* that is used in food fermentations (Supplementary Table 7). Mung bean sprout brand A harbored the spoilage associated bacteria, *Leuconostoc* and *Klebsiella*, as well as *Comamonas* genera that were not observed in brand B (Supplementary Table 7). Mung bean sprout brand B harbored six genera that we did not observe in brand A including *Sphingomonas*, *Chryseobacterium*, *Lelliottia*, *Xanthomonas*, *Herbaspirillum*, and *Janthinobacterium* (Supplementary Table 7). The cilantro samples harbored a unique cohort of *Burkholderiales* including *Massilia*, *Duganella*, *Variovorax*, and *Acidovorax* species (Supplementary Table 7). Other taxa unique to cilantro were *Alkanindiges*, *Flavobacterium*, *Pedobacter*, *Shewanella*, *Paracoccus*, *Methylotenera*, and *Aquaspirillum* (Supplementary Table 7). *Carnobacterium* and *Psychrobacter* were unique to smoked salmon and cilantro.

## Discussion

Identifying baseline microbiomes in foods is an important first step to observing bacteria that co-enrich with foodborne pathogens. Since the resident bacteria will resuscitate simultaneously with foodborne pathogens they may also compete for nutrients during the non-selective and selective culture enrichment steps and hamper pathogen detection. Thus, understanding these microbiome profiles may provide knowledge on improving culture enrichment methods. Subsequent to this study we incorporated heterotrophic plate counting into our workflow since it is possible and likely that some of the biomass we observed using 16S rRNA gene amplicon sequencing is from non-viable bacteria. Our results revealed bacterial loads of 4 log CFU/gm in smoked salmon, 5 log CFU/gm in garam masala spice mix, and 7–8 log CFU/gm in cilantro (unpublished data). Our findings support a previous microbiome study that demonstrated commodity driven bacterial diversity in conventionally and organically grown fresh fruits and vegetables (Leff and Fierer, 2013). For example, sprouts, spinach, lettuce, tomatoes, peppers, and strawberries, harbored high levels of *Enterobacteriaceae* and the microbiomes of apples, grapes, peaches, and mushrooms were predominated by *Actinobacteria*, *Firmicutes*, *Bacteroidetes*, and *Proteobacteria* (Leff and Fierer, 2013). The microbial community profiling in this study also expands our knowledge about bacteria that may hamper pathogen detection or impact shelf life (Daquigan et al., 2016; Ottesen et al., 2016; Grim et al., 2017).

Examining food microbiomes in a culture independent manner provides a comprehensive characterization of resident microbial communities and their dynamics that can be utilized in many ways in food-related pursuits. For example, microbiome characterization is extensively used in the study of fermented foods, to help bring about enhancements in the finished product. Detection of adulteration or contamination, whether it be economically based or accidental exposure to a microbial pathogen, and the study of spoilage events are other areas where food microbiome research is growing. Historically, the assessment of quality and safety of food commodities, such as spices, was achieved using standard microbiological methods such as aerobic plate counts, and biochemical phenotypic typing assays to quantify and identify target bacteria (Pafumi, 1986; Satchell et al., 1989; Garcia et al., 2001; Sagoo et al., 2009; Sospedra et al., 2010). In this study, we examined the bacterial microbiomes associated with several food commodities, to establish baseline data, which will be available publicly at the FDA MetagenomeTrakr bioproject at NCBI.

In our masala spice mix samples, we observed a high prevalence of *Enterobacter* and *Bacillus* species with 16S rRNA gene sequencing, in agreement with previous spice investigations that measured total bacterial loads ranging from 10^2^ to 10^7^ CFU/gm, with a predominance of *Enterobacter* and *Bacillus* (Pafumi, 1986; Satchell et al., 1989; Garcia et al., 2001; Sagoo et al., 2009; Sospedra et al., 2010). A survey of black peppercorns, white peppercorns, coriander, and fennel seeds identified *Enterobacter cloacae* as the most common *Enterobacteriaceae* member, which is consistent with our findings where proportional abundances of *Enterobacter* species ranged from 11 to 23% with additional abundances (7–13%) of unidentified *Enterobacteriaceae* that are likely *Enterobacter* species (Satchell et al., 1989). The high bacterial diversity and number of unique species (19) we observed in masala spice was expected since the spice mixtures tested were comprised of as many as 17 different ingredients and most published studies were of individual spices. Factors noted as likely contributors to variations in bacterial loads in spices included pre- and post-harvest practices and packaging methods. For example, a review of spices from Mexican markets that were non-packaged or packaged in polyethylene bags or glass containers, revealed relatively low levels of total and fecal coliform counts in the glass and non-packaged spices while 70% of the spices packaged in polyethylene bags contained 10^3^ CFU/gm of these bacteria (Garcia et al., 2001). Spice harvest practices such as laying spices on the ground to dry and the necessity of human handling are likely responsible for the *Staphylococcal* abundances observed in this study as was previously shown in a survey of herbs and spices collected in Spain (Sospedra et al., 2010). Although in our study we observed *Staphylococcus sciuri* which is a commensal animal-associated bacteria suggesting animals as a route of adulteration (Nemeghaire et al., 2014). An advantage of 16S rRNA gene sequencing over microbiological testing is the identification of the entirety of a much more diverse population that includes organisms such as *Pseudomonas*, *Pantoea*, *Acinetobacter*, and *Serratia* that may be overlooked using standard microbiological culture methods since these methods tend to select for particular bacterial populations.

We extended upon our previous work with cilantro in this study in two ways: first, we employed paired end sequencing using the Illumina MiSeq which is much more robust than the 454-pyrosequencing used in our original study and second, we sequenced samples from two points in the supply chain, post-harvest (MDARD) and retail (Jarvis et al., 2015). We confirmed our original microbiome composition of mostly *Proteobacteria* with low levels of *Firmicutes* and *Actinobacteria.* We also observed differences between MDARD and retail cilantro with the retail samples containing higher proportional abundances of *Flavobacterium* (34%), commonly recovered from terrestrial (soil, rhizosphere, and phyllosphere) and aquatic environments (Kolton et al., 2013). A study of niche adaptation among *Flavobacterium* found evidence that terrestrial species have adapted to rhizosphere and phyllosphere niches where they can preferentially metabolize plant carbohydrates while aquatic species had larger genomes with higher ratios of peptide and protein utilization genes (Kolton et al., 2013). Most of the species we observed in the retail cilantro were from aquatic environments. Further research is required to identify handling practices that may have contributed to the higher abundances of *Flavobacterium* in CILSB retail samples, which could also be indicative of a more advanced state of spoilage in these samples since they were collected at a later stage in the farm to fork continuum than the MDARD cilantro (Blackburn, 2006; Wareing et al., 2010).

The cucumbers each harbored similar bacterial community members at different proportional abundances. Since cucumbers grow on the ground, it is likely that the local soil and rhizosphere bacteria contribute heavily to the cucumber microbiota. The predominance of *Proteobacteria* on all cucumbers tested in our study is corroborated by a study specific to cucumber rhizospheres, and other studies focused on the bacterial diversity of soil and rhizosphere biology (Spain et al., 2009; Mendes et al., 2013; Tian and Gao, 2014). Our identification of *Acinetobacter* on cucumbers and in the masala spice mixes, at much lower abundances, is notable since some species are known to cause nosocomial infections. Species of *Acinetobacter* are also known to harbor genes conferring antimicrobial resistance and, in a recent study focused on screening ready-to-eat produce (lettuce, apples, pears, bananas, and strawberries), *Acinetobacter* was isolated from 77.9% of the samples screened (Carvalheira et al., 2017). Furthermore, antimicrobial susceptibility screening of the *Acinetobacter* isolated from lettuce and fruits revealed resistance to 14 antimicrobial agents, including colistin, in 13.3% of the strains (Carvalheira et al., 2017). The notion of vegetables as transmission vehicles of *Acinetobacter* is not new as a vegetable study in 1999 identified antimicrobial resistant species of the *A. calcoaceticus–A. baumannii complex* in 17% of the vegetables tested (Berlau et al., 1999). The presence of *Acinetobacter* in cucumber microbiomes is also an indicator of decomposition since they are known to cause food spoilage. The cucumbers in our study also harbored the highest proportional abundances of gram-positive soil associated *Actinobacteria* (8–31%) such as *Curtobacterium*, *Microbacterium*, and *Arthrobacter* among the foods sequenced. Finally, the presence of *Paenibacillus* at an abundance of 31% is CUC5 is notable from a food safety perspective as members of this genera have been linked to inhibiting *Salmonella* growth (Lorentz et al., 2006; Luo et al., 2013; Allard et al., 2014; Knolhoff et al., 2015; Sprando et al., 2017). The FDA relies on culture methods to recover *Salmonella* from food and it is possible that *Paenibacillus* could hamper *Salmonella* recovery from contaminated commodities during outbreak investigations.

Our findings with the SPA mung bean sprout sample that contained high proportional abundances of *Leuconostoc* and *Lactococcus*, suggests that these sprouts were in an advanced stage of spoilage, compared to SPB. This is noteworthy, since we did not observe visible compositional changes to either mung bean sprout sample. Our analysis identified a mixture of *Leuconostoc* OTUs at proportional abundances reaching 50% which likely represent one of three *L*. *gelidum* subspecies; *L*. *gelidum* subsp. *gelidum*, *L*. *gelidum* subsp. *gasicomitatum*, and *L*. *gelidum* subsp. *aenigmaticum* that are associated with spoilage in modified-atmosphere-packaged (MAP) foods (Rahkila et al., 2015). These psychrotrophic lactic acid bacteria have been associated with spoilage in packaged meat and vegetable products (Rahkila et al., 2015). *L*. *citreum*, another lactic acid bacteria, was also presumptively identified in SPA at proportional abundances of 14%. Interestingly, both *L*. *gelidum* and *L*. *citreum* are favorable members of food microbial communities since they produce bacteriocins active against *Listeria monocytogenes* (Hastings et al., 1991; Van Belkum and Stiles, 1995; Pujato et al., 2014). The SPA samples also harbored high proportional abundances of *Lactococcus* species (29%), presumptively identified as *L*. *raffinolactis* (13%) and *L*. *piscium* (13%). *L*. *piscium* has been isolated from vacuum packaged refrigerated beef (Sakala et al., 2002) and it is the only *Lactococcus* species associated with food spoilage in MAP packaged beef and vegetables (Rahkila et al., 2012; Pothakos et al., 2014). The second set of mung bean sprout samples, SPB, had a similar microbiota composition to the one defined by Margot et al. (2016) and Leff and Fierer (2013) comprised mostly *Proteobacteria*. However, SPB contained predominantly *Enterobacter* species while the microbiota in Margot et al. (2016) had a predominance of *Janthinobacterium* and the bean sprouts sequence by Leff and Fierer (2013) harbored mostly *Pantoea*.

Our microbiome observations in smoked salmon revealed a predominance of *Lactobacillus*, *Brochothrix*, *Leuconostoc*, and *Carnobacterium* species, indicative of spoilage which was not surprising since these samples had been opened for at least 1 week by the time our laboratory received them and spoilage of smoked salmon happens rather rapidly even at refrigerated conditions (Chaillou et al., 2015). These species parallel those found in a microbiological study where the smoking process and type of packaging were implicated in shaping the spoilage-associated microbiota in smoked salmon (Leroi et al., 1998; Emborg et al., 2002; Olofsson et al., 2007; Mace et al., 2013). A recent 16S rRNA gene sequencing comparison of fresh and spoiled smoked salmon corroborate our findings of low species richness (Chaillou et al., 2015). Chaillou et al. (2015) reported fluctuations in alpha diversity from 189 ± 58 operational taxonomic units (OTUs) in fresh seafood to 27 ± 12 OTUs in spoiled products. The alpha diversity of the observed species in our smoked salmon samples were 180 (E1) and 181(E2), 141 (F1) and 132 (F2), and 89 in both SmkSalD replicates, indicating that they may have been at different stages of spoilage even though all three samples harbored high abundances of spoilage associated bacteria. However, the types of spoilage associated bacteria in our study varied between samples such that SmkSalD contained the highest levels of *Lactobacillus*, SmkSalE harbored high levels of *Psychrobacter*, and *Brochothrix* and SmkSalF harbored a predominance of *Serratia*. Chaillou et al. (2015) identified *Brochothrix thermosphacta* as the most ubiquitous and abundant species among all of the meat and seafood they tested.

All of the foods in this study harbored microbiome profiles that would eventually lead to bacterial spoilage. For example, the presence of *Flavobacterium*, *Erwinia*, *Pantoea*, *Acinetobacter*, and *Pseudomonas* species in all plant food microbiomes supports the notion that spoilage can be predicted by assessing food microbiome profiles since they are all known to contribute to food spoilage (Blackburn, 2006; Wareing et al., 2010; Parlapani et al., 2013; Gu et al., 2018). Additionally, psychrotolerant species such as *Acinetobacter*, *Pseudomonas*, *Psychrobacter* and others, that are capable of surviving and growing at cold temperatures will increase in numbers after harvest in cucumbers, cilantro, and mung bean sprouts that have cold storage requirements. The high abundances of spoilage associated *Enterobacteriaceae* (*Erwinia* and *Pantoea*), *Pseudomonas*, and *Acinetobacter* in mung bean sprout brand B and *Leuconostoc* and *Lactococcus* in brand A demonstrate different stages or types of bacterial spoilage in MAP foods. We also observed lactic acid bacteria with known biopreservative functions such as *Lactobacillus* and *Carnobacterium*. These genera are known to produce antimicrobial substances (organic acids, peroxides, and antimicrobial peptides) and are purposely added to some foods to preserve and protect against spoilage and pathogenic bacteria (Calix-Lara et al., 2014; Spanu et al., 2017). Not surprisingly the masala spices which had the highest alpha-diversity among the foods we sequenced also harbored the most diverse population of spoilage associated bacteria and they were the only food that contained *Bacillus* species.

Sequencing food microbiomes reveals key features about food safety and quality. For example, in foods with highly diverse and complex microbiomes such as cilantro, studies have shown that pathogen detection is hampered when closely related species are present (Grim et al., 2017). A spinach microbiome study corroborated this for *Escherichia coli* detection (Leonard et al., 2016). Our findings of various food spoilage bacteria in the mung bean sprouts and smoked salmon demonstrate the ability to assess food quality. The identification of clinically significant bacteria such as *Staphylococcus* and *Acinetobacter* suggest that food could play a role in transmission of organisms from the environment to humans. Finally, there is the notion that the microbiomes of foods that are consumed raw could have favorable contributions to our gut microbiomes. Supporting these hypotheses by defining core microbiomes of foods derived from different ecological niches will provide information that can be used to assess food health in a similar fashion to the application of human microbiome findings to human health. In this regard, we can also begin to understand the impact that food microbiomes have on gut health.

## Author Contributions

KJ, CG, AO, PR, and PM designed the project. KJ, ND, CG, PM, LH, and JM conducted the experiments. JW performed the bioinformatic analyses. All authors read and revised the manuscript.

## Conflict of Interest Statement

JW is founder of Resphera Biosciences and has an equity position in the company. The remaining authors declare that the research was conducted in the absence of any commercial or financial relationships that could be construed as a potential conflict of interest.
