# Supplementary material for: Microbiomes Associated With Foods From Plant and Animal Sources
Source: Front Microbiol. 2018 Oct 23;9:2540. doi: 10.3389/fmicb.2018.02540 (PMC6206262; doi:10.3389/fmicb.2018.02540)
Supplement: Supplementary file 2 [file Presentation_1.PPTX]

## Slide 1
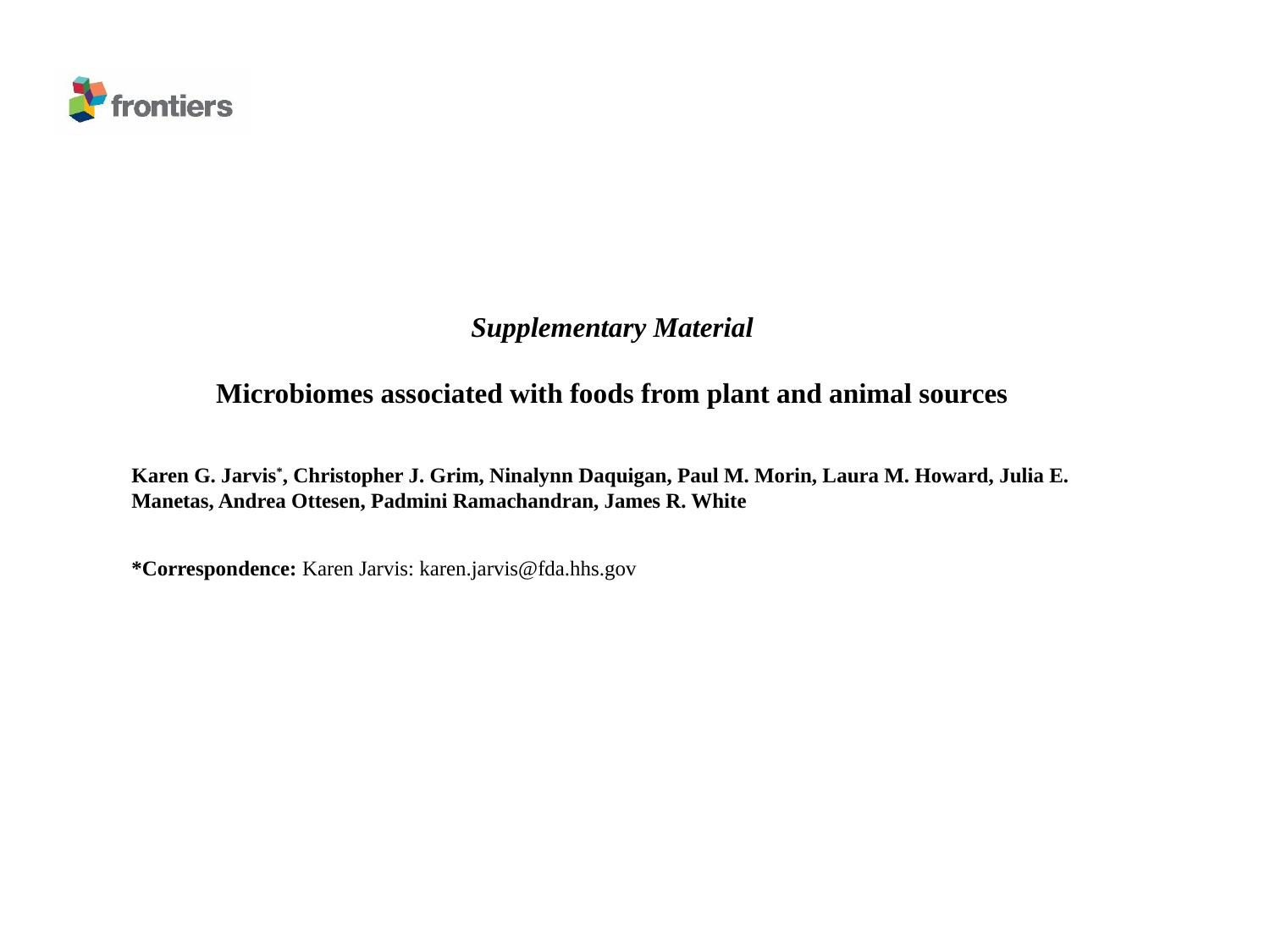

Supplementary Material
Microbiomes associated with foods from plant and animal sources
Karen G. Jarvis*, Christopher J. Grim, Ninalynn Daquigan, Paul M. Morin, Laura M. Howard, Julia E. Manetas, Andrea Ottesen, Padmini Ramachandran, James R. White
*Correspondence: Karen Jarvis: karen.jarvis@fda.hhs.gov

## Slide 2
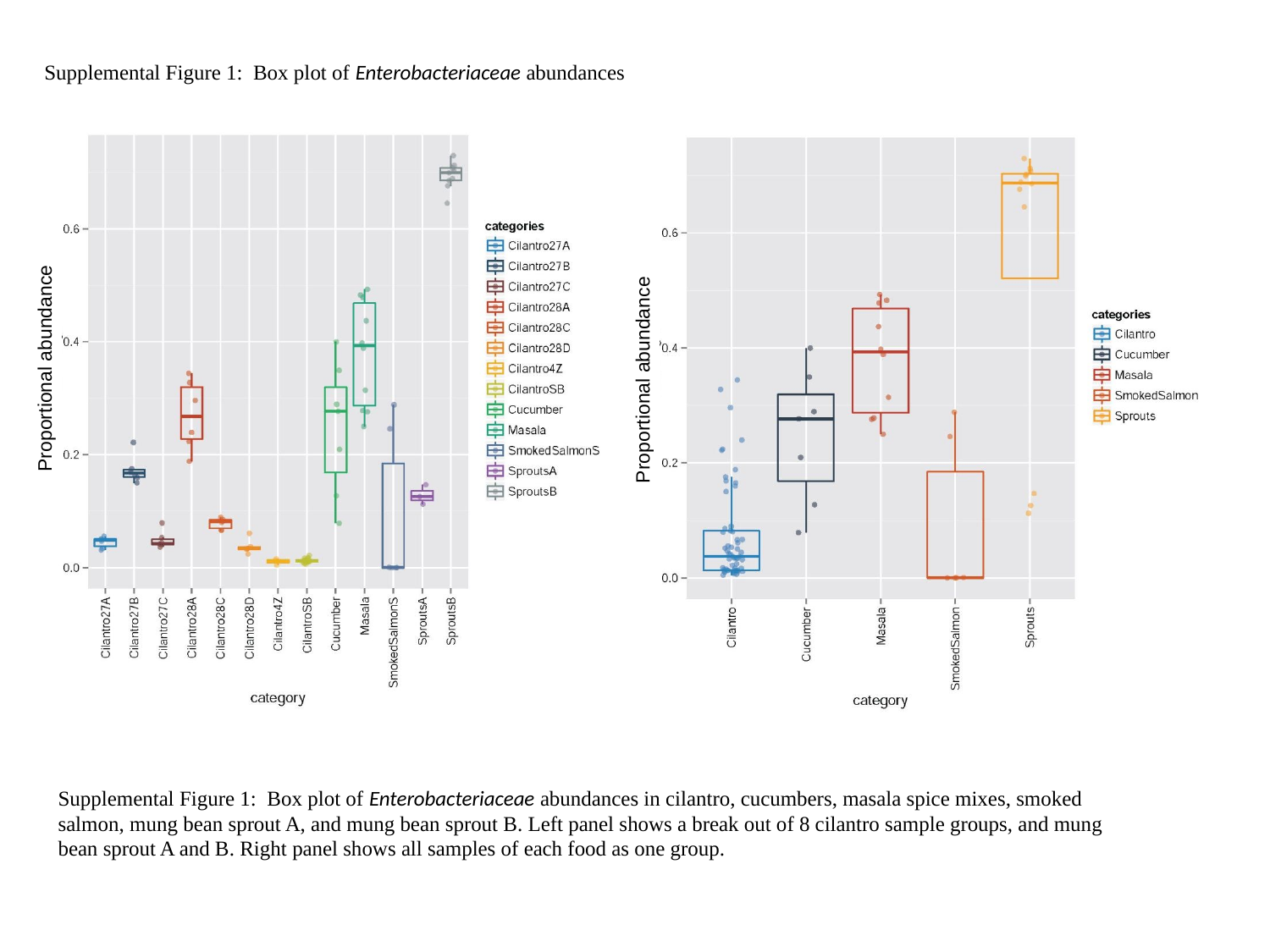

Supplemental Figure 1: Box plot of Enterobacteriaceae abundances
Proportional abundance
Proportional abundance
Supplemental Figure 1: Box plot of Enterobacteriaceae abundances in cilantro, cucumbers, masala spice mixes, smoked salmon, mung bean sprout A, and mung bean sprout B. Left panel shows a break out of 8 cilantro sample groups, and mung bean sprout A and B. Right panel shows all samples of each food as one group.

## Slide 3
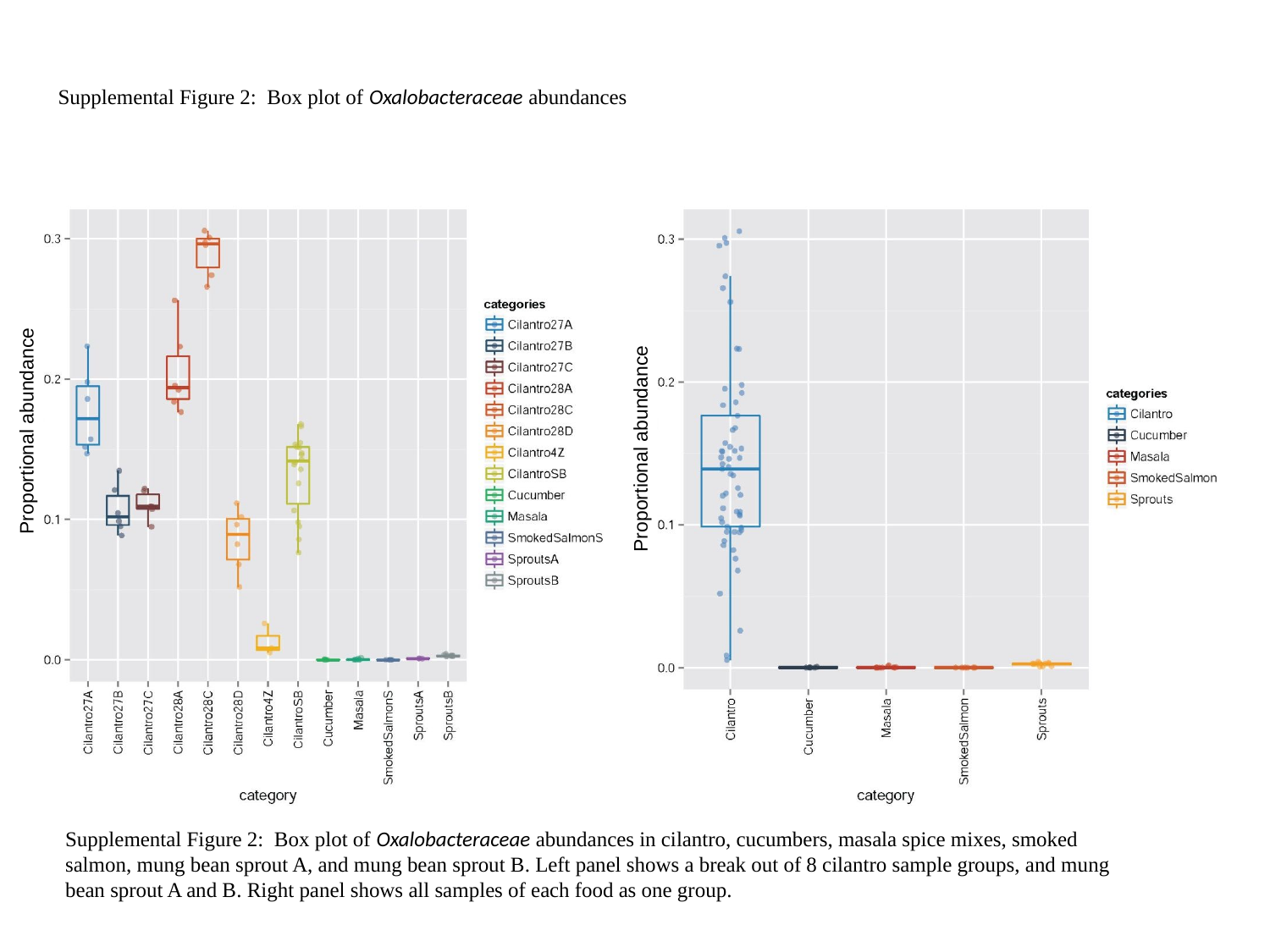

Supplemental Figure 2: Box plot of Oxalobacteraceae abundances
Proportional abundance
Proportional abundance
Supplemental Figure 2: Box plot of Oxalobacteraceae abundances in cilantro, cucumbers, masala spice mixes, smoked salmon, mung bean sprout A, and mung bean sprout B. Left panel shows a break out of 8 cilantro sample groups, and mung bean sprout A and B. Right panel shows all samples of each food as one group.

## Slide 4
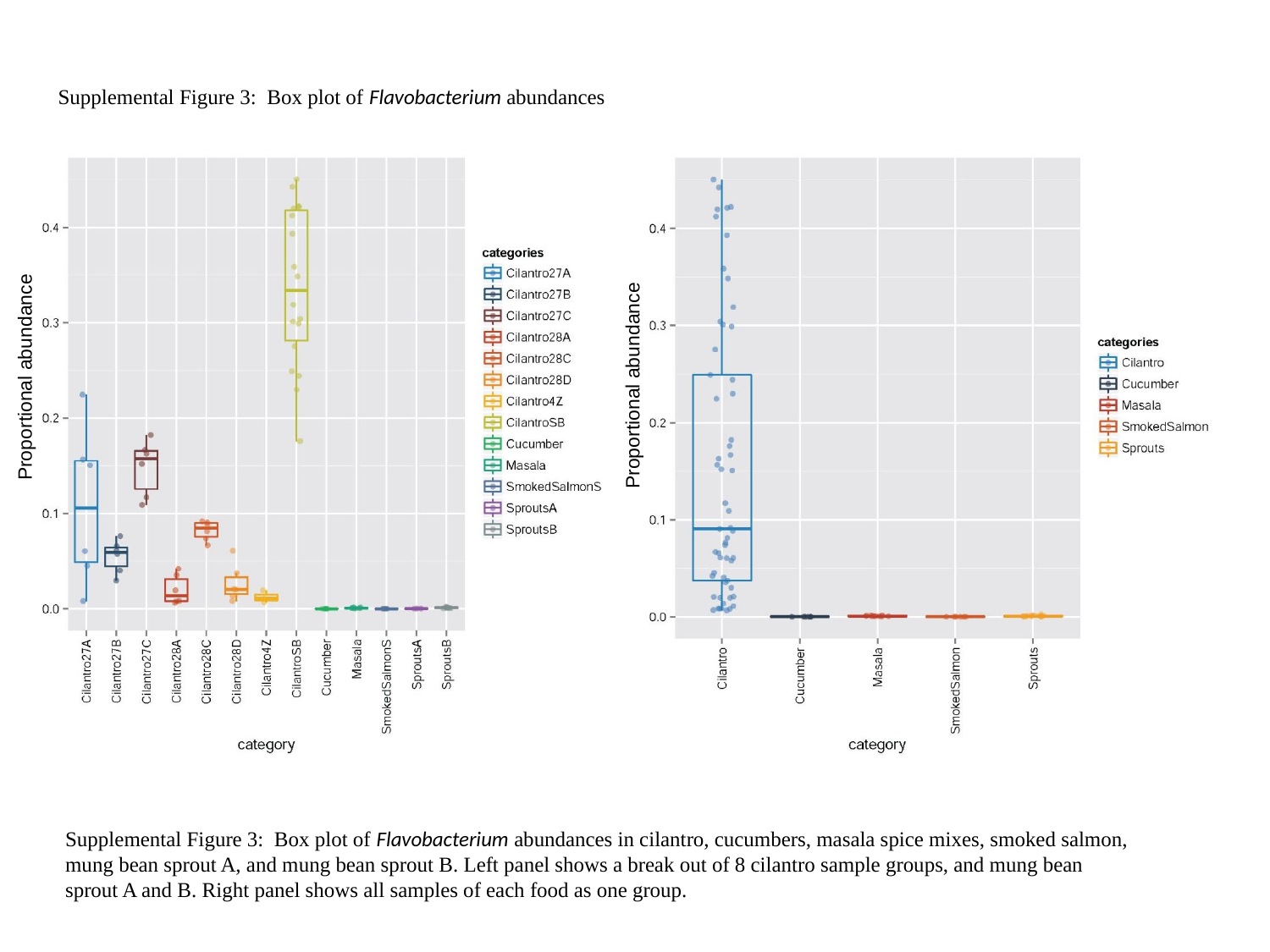

Supplemental Figure 3: Box plot of Flavobacterium abundances
Proportional abundance
Proportional abundance
Supplemental Figure 3: Box plot of Flavobacterium abundances in cilantro, cucumbers, masala spice mixes, smoked salmon, mung bean sprout A, and mung bean sprout B. Left panel shows a break out of 8 cilantro sample groups, and mung bean sprout A and B. Right panel shows all samples of each food as one group.

## Slide 5
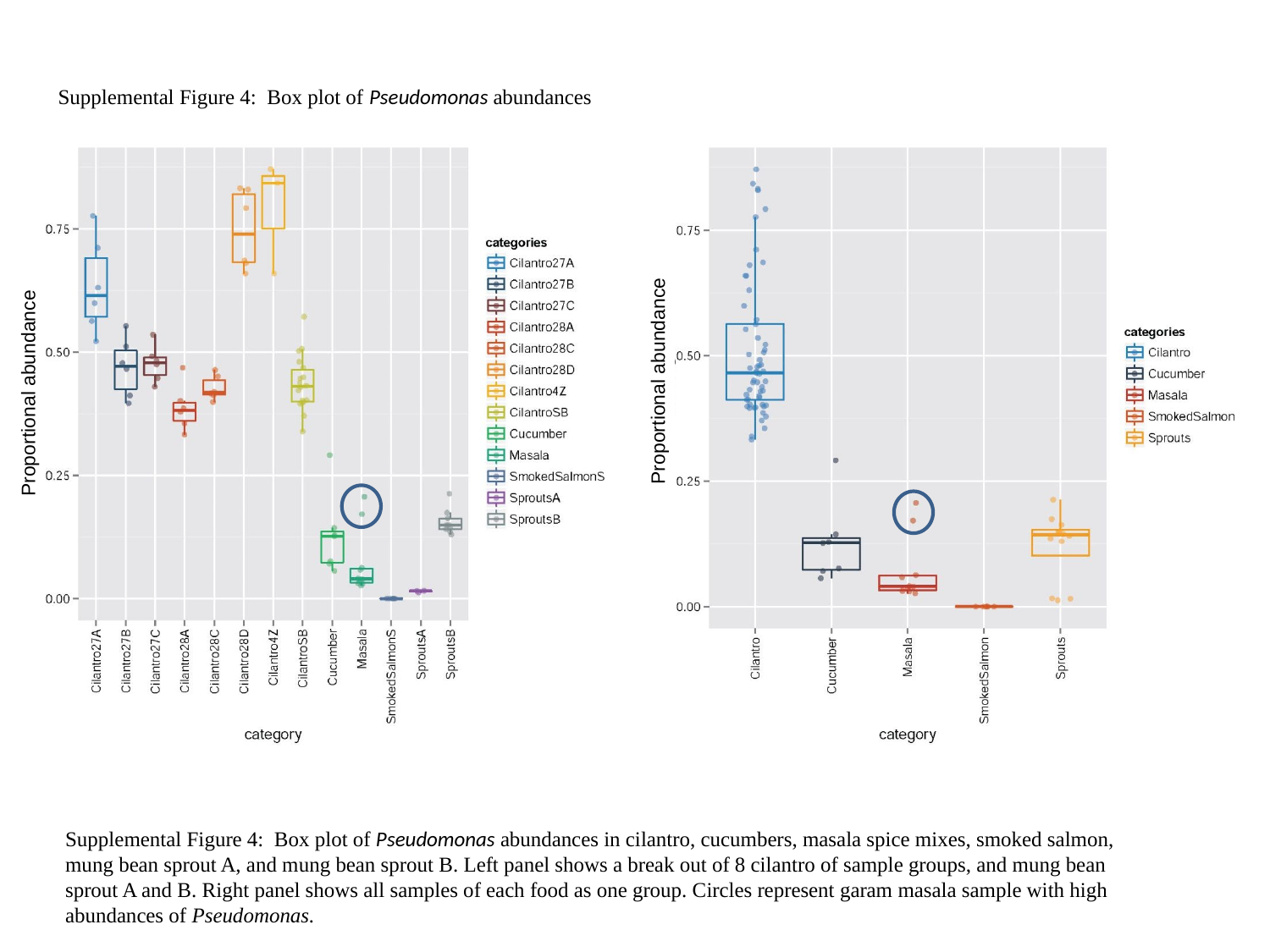

Supplemental Figure 4: Box plot of Pseudomonas abundances
Proportional abundance
Proportional abundance
Supplemental Figure 4: Box plot of Pseudomonas abundances in cilantro, cucumbers, masala spice mixes, smoked salmon, mung bean sprout A, and mung bean sprout B. Left panel shows a break out of 8 cilantro of sample groups, and mung bean sprout A and B. Right panel shows all samples of each food as one group. Circles represent garam masala sample with high abundances of Pseudomonas.

## Slide 6
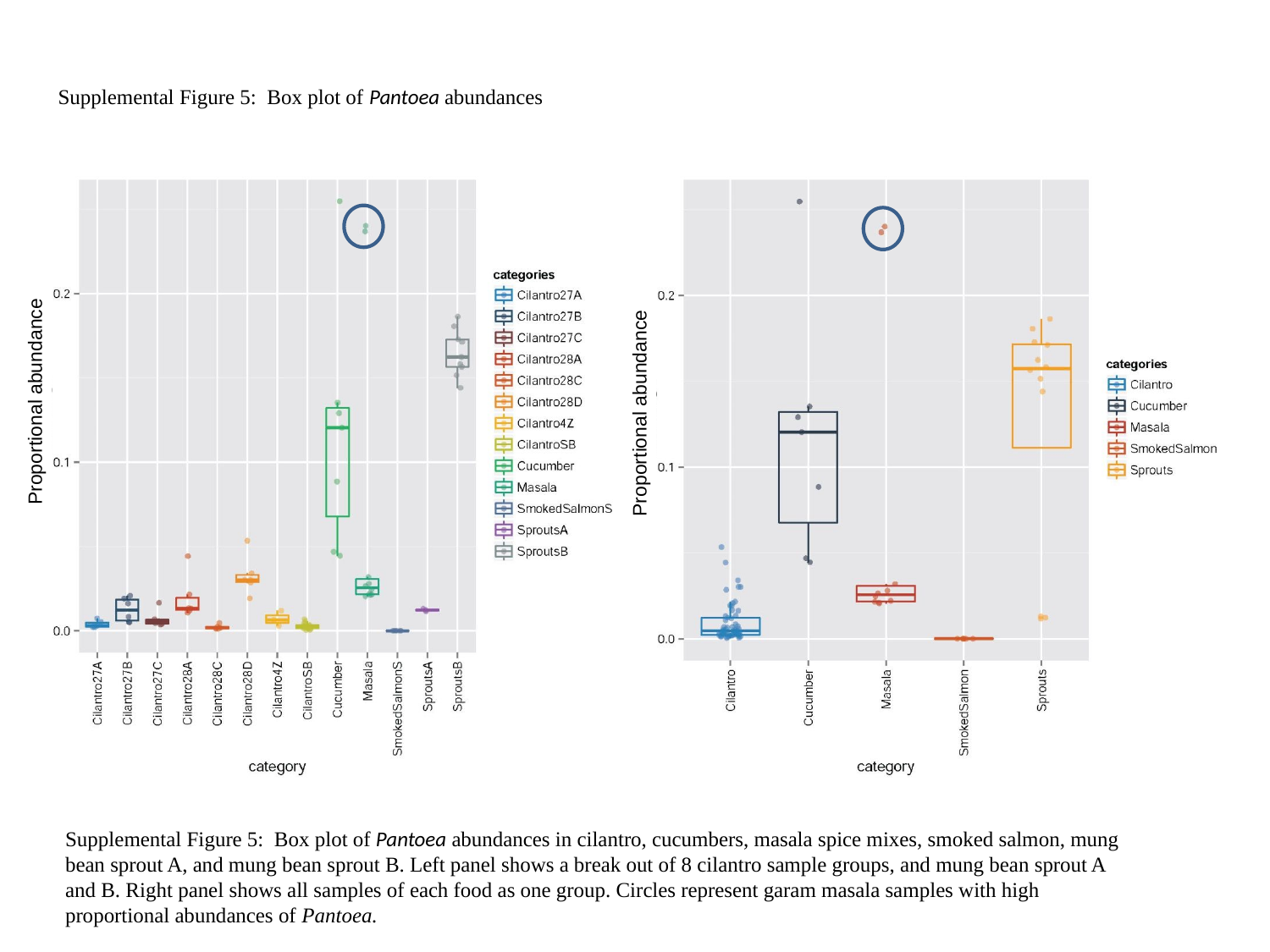

Supplemental Figure 5: Box plot of Pantoea abundances
Proportional abundance
Proportional abundance
Supplemental Figure 5: Box plot of Pantoea abundances in cilantro, cucumbers, masala spice mixes, smoked salmon, mung bean sprout A, and mung bean sprout B. Left panel shows a break out of 8 cilantro sample groups, and mung bean sprout A and B. Right panel shows all samples of each food as one group. Circles represent garam masala samples with high proportional abundances of Pantoea.
